# The cost-saving effect of centralized histological reviews with soft tissue and visceral sarcomas, GIST, and desmoid tumors: The experiences of the pathologists of the French Sarcoma Group

**DOI:** 10.1371/journal.pone.0193330

**Published:** 2018-04-05

**Authors:** Lionel Perrier, Pauline Rascle, Magali Morelle, Maud Toulmonde, Dominique Ranchere Vince, Axel Le Cesne, Philippe Terrier, Agnès Neuville, Pierre Meeus, Fadila Farsi, Françoise Ducimetière, Jean-Yves Blay, Isabelle Ray Coquard, Jean-Michel Coindre

**Affiliations:** 1 University of Lyon, Léon Bérard Cancer Centre, GATE L-SE UMR 5824, Lyon, France; 2 Direction of Clinical Research and Innovation, DRCI, Léon Bérard Cancer Centre, Lyon, France; 3 Department of Medicine, Institut Bergonié, Bordeaux, France; 4 Department of Anatomopathology, Cancer Centre Léon Bérard, Lyon, France; 5 Department of Medecine, Institut Gustave Roussy, Villejuif, France; 6 Department of Pathology, Gustave Roussy, Villejuif, France; 7 Department of Anatomopathology, Institut Bergonié, Bordeaux, France; 8 Department of Surgery, Cancer Centre Léon Bérard, Lyon, France; 9 Réseau Espace Santé Cancer Rhône-Alpes, Lyon, France; 10 Santé-Individu-société EA-INSERM 4129, Cancer centre Léon Bérard, Lyon, France; 11 Department of Medicine, Cancer Centre Léon Bérard, Lyon, France; 12 University Victor Ségalen, Bordeaux, France; Universite de Nantes, FRANCE

## Abstract

**Objective:**

This study examined the types of discordance occurring in the diagnosis of soft tissue and visceral sarcomas, gastrointestinal stromal tumors (GIST), and desmoid tumors, as well as the economic impact of diagnostic discrepancies.

**Methods:**

We carried out a retrospective, multicenter analysis using prospectively implemented databases performed on a cohort of patients within the French RRePS network in 2010. Diagnoses were deemed to be discordant based on the 2013 World Health Organization (WHO) classification. Predictive factors of discordant diagnoses were explored. A decision tree was used to assess the expected costs of two strategies of disease management: one based on revised diagnoses after centralized histological review (option 1), the other on diagnoses without centralized review (option 2). Both were defined based on the patient and the disease characteristics, according to national or international guidelines. The time horizon was 12 months and the perspective of the French National Health Insurance (NHI) was retained. Costs were expressed in Euros for 2013. Sensitivity analyses were performed using low and high scenarios that included ± 20% estimates for cost.

**Results:**

A total of 2,425 patients were included. Three hundred forty-one patients (14%) had received discordant diagnoses. These discordances were determined to mainly be benign tumors diagnosed as sarcomas (n = 124), or non-sarcoma malignant tumors diagnosed as sarcomas (n = 77). The probability of discordance was higher for a final diagnosis of desmoid tumors when compared to liposarcomas (odds ratio = 5.1; 95%CI [2.6–10.4]). The expected costs per patient for the base-case analysis (low- and high-case scenarios) amounted to €8,791 (€7,033 and €10,549, respectively) for option 1 and €8,904 (€7,057 and €10,750, respectively) for option 2.

**Conclusions:**

Our findings highlight misdiagnoses of sarcomas, which were found to most often be confused with benign tumors. Centralized histological reviews are likely to provide cost-savings for the French NHI.

## Introduction

Discordant diagnoses are known to occur for most malignancies, with a wide range of discordance rates due mainly to the use of variable definitions for discordance, the nature of the histological review used (i.e., a second opinion request *vs*. systematic review), and the tumor type [1–7]. Nevertheless, the use of a histological review to improve the accuracy of histological diagnosis in oncology, particularly with rare cancers, has recently received considerable attention due to increased efforts to enhance institutional performance and to reduce medical errors [4, 6, 8–12]. In light of the value of histological review, policies in support of its use in oncology have become more prevalent [13, 14].

However, significant obstacles remain to effectively implement the histological review process, including its cost in terms of the pathologist’s time and a generally low or absent financial remuneration by third-party payers. Nevertheless, healthcare institutions have started to develop local policies to perform histological reviews [5, 6]. Most of these institutions recommend referral to a tertiary care center to review the initial diagnosis [3, 6]. Indeed, mandatory histological reviews might be more important for sarcoma patients, as studies have reported discordance rates from 27% to 42% in this population [12, 15]. Sarcomas are malignant tumors that develop in soft tissues, bone, skin, and internal organs. The large majority of soft tissue tumors are benign, and these benign lesions are two orders of magnitude more common than malignant soft tissue lesions. Moreover, sarcomas include more than 50 histological subtypes, all of which are rare tumors. Thus, making an accurate diagnosis can prove difficult for non-specialized pathologists [16–18].

Due to the high discordance rates in the diagnosis of sarcoma, with potential detrimental effects on medical decision-making, and the findings of the Connective Tissue Cancer Network (CONTICANET), the French National Cancer Institute has implemented a national network dedicated to histological review of sarcomas, GIST, and desmoid tumors [15, 16]. The RRePS (Réseau de Référence en Pathologie des Sarcomes) network was created in January 2010 and it monitors the approximately 4,000 patients diagnosed annually with sarcoma out France’s population of the 64.6 million inhabitants [19, 20].

There have been few studies regarding the possible demographic, pathological, or clinical characteristics associated with clinically-significant diagnosis revisions. This paucity of information prompted us to report regarding discordant diagnoses that were observed during the first year after establishment of the RRePS network [21]. As an extension of this preliminary study, the objectives of our present study were (i) to investigate the types of discordances that occur; (ii) to determine predictive factors for non-concordance in regard to sarcomas, gastrointestinal stromal tumors (GIST), and desmoid tumors; and (iii) to compare the cost of disease management based on revised diagnoses after centralized histological reviews and disease management based on diagnoses before review.

## Methods

### Patient inclusion

In 2010, all of the patients with a diagnosis or suspicion of a primary sarcoma, GIST, or desmoid tumor (soft tissue or visceral) and with a histological review performed by the RRePS network were included. The patients were managed according to the ethical principles for medical research involving human subjects as described in the Declaration of Helsinki. Moreover, the present study received approval from the Consultative Committee on Data Processing Regarding Research in the Field of the Health (CCTIRS, n°12576Bis) and the National Committee for Protection of Personal Data (CNIL, n°910390). Data are from the Study "Evaluation économique des relectures dans les sarcomes" whose authors may be contacted at the Cancer Centre Léon Bérard, Lyon, France.

### Histological review procedure within the RRePS network

The network comprises a total of 22 French pathology centers, including three coordinating centers (the Institut Bergonié in Bordeaux, the Centre Léon Bérard in Lyon, and the Institut Gustave Roussy in Villejuif) and 19 referring centers throughout France. Each center reviewed cases from the corresponding area using systematic molecular testing for every tumor that was suspected to have a specific genomic abnormality (e.g., *KIT* and *PDGFRA* mutations for GIST, *CTNNB1* mutation for desmoid tumors, specific translocations for sarcomas with a recurrent translocation, and *MDM2/CDK4* amplifications for atypical/well-differentiated and dedifferentiated liposarcomas). In addition, immunohistochemistry was used when required. Furthermore, every tumor without a specific abnormality (i.e., a genomic abnormality, HHV8-positive Kaposi sarcoma, or KIT- and DOG1-positive GIST [via immunohistochemistry]) was systematically reviewed by a coordinating center or during a multi-head histological session (one full day each month). Local pathologists either sent cases to be reviewed for second opinions when diagnostic difficulties were encountered or for systematic review according to the French National Cancer Institute’s (NCI; Institut National du Cancer [INCa]) recommendations. Internal network refers to a second opinion between pathologists of the RRePS network. For each case examined, data related to the patient and the tumor characteristics were compiled in a shared database (https://rreps.sarcomabcb.org).

### Definition of concordant and discordant diagnoses

Diagnoses were considered to be concordant only when the final diagnosis belonged to the same main category of tumor as the initial diagnosis, as defined by the World Health Organization (WHO) Classification of Tumors of Soft Tissue and Bone [18]. From the start of the RRePS network, we anticipated the main changes to the WHO2002 classification and we largely followed the WHO2013 classification. Diagnoses that did not fit these criteria were considered to be discordant. The categories were: sarcomas, intermediate malignant tumors, GIST, desmoid tumors, non-sarcoma malignant tumors, and benign tumors. GIST and desmoid tumors were distinguished based on their specific therapeutic indications. Notably, histopathological grade and/or subtype differences were not taken into account for our analysis. Also, in the event that the pathologists proposed several diagnoses, the first one was retained for the evaluation of concordance.

### Statistical analysis

Chi-square and Mann-Whitney-Wilcoxon tests were used to compare the patient and the tumor characteristics between the concordant and the discordant groups. Logistic regression analysis with backward variable selection was performed to determine the set of factors that best predict discordance. Patients with a final diagnosis of a benign tumor or a non-sarcoma malignant tumor where excluded from the regression analysis as they were clearly associated with a discordance.

### Cost analysis

A decision tree was generated in order to estimate the expected costs of both options: disease management based on revised diagnoses after centralized histological review (option 1) and disease management based on diagnoses before review (i.e., without a histological review, and referred to as option 2). The French National Health Insurance (NHI) perspective was retained. Five steps were taken:

Step 1 Identification of the resources used. The initial treatments (i.e., work-up for tumor extension, surgery, chemotherapy, radiotherapy, and other treatments), as well as the one year follow-up period (post-treatment surveillance), were considered. Disease management based on option 1 and option 2 was defined for each patient with a discordant diagnosis identified after a histological review performed within the RRePS network. Disease management was assessed by three clinicians, according to the characteristics of the patient and of the disease using national or international guidelines. More specifically, the recommendations for clinical practice from the 2012 European Society for Medical Oncology and from the 2006 National Anti-Cancer Federation were used for sarcoma and GIST. Guidelines from the French National Cancer Institute or by default from territorial cancer networks (e.g., Réseau Espace Santé-Cancer, Rhône-Alpes) were also taken into account for the other categories of tumors. The data used to define therapeutic decisions are reported in S1 File.
Step 2 Cost assessment. The costs of hospitalizations were assessed based on the Diagnosis-related groups (DRGs) tariffs. External consultations and broader examinations (e.g., magnetic resonance imaging) were evaluated using the General nomenclature of professional act (NGAP) and the Classification Commune des Actes Médicaux (CCAM). The costs of pathology examinations were based on the Nomenclature des Actes de Biologie Médicale (NABM). Costs were calculated for each patient with a discordant diagnosis identified following a histological review performed within the RRePS network of disease management based on (i) revised diagnoses after centralized histological review (option 1) and (ii) disease management based on diagnoses before review (option 2). The cost per patient with concordant diagnoses was assessed based on diagnoses before review. For option 1, additional costs were included: the cost of histological review (based on the publication of Lapeyrere et al. [8]) and the cost of the RRePS network organization. The cost of the RRePS network organization was calculated as follows: the annual budget awarded to the RRePS network by the French National Cancer Institute (in 2010) divided by the number of patients included in the network (in 2010). All costs were expressed in Euros, using costs and unit prices from 2013. Costs were not discounted due to the short time horizon.
Step 3 Probability calculations. Probabilities were based on all of the patients who underwent histological review in the RRePS network in 2010.
Step 4 Expected costs calculation. Expected costs were calculated for both options using MS Excel 2007^®^.
Step 5 Uncertainty assessment. Sensitivity analyses were performed using low and high scenarios, which included estimates ± 20% for all of the cost parameters from the base-case. Additional one-way sensitivity analyses were carried out on the cost of histological review and the cost of the RRePS network organization using TreePlan ToolKit Classic^®^.

## Results

### Patient inclusion

**Fig 1:**
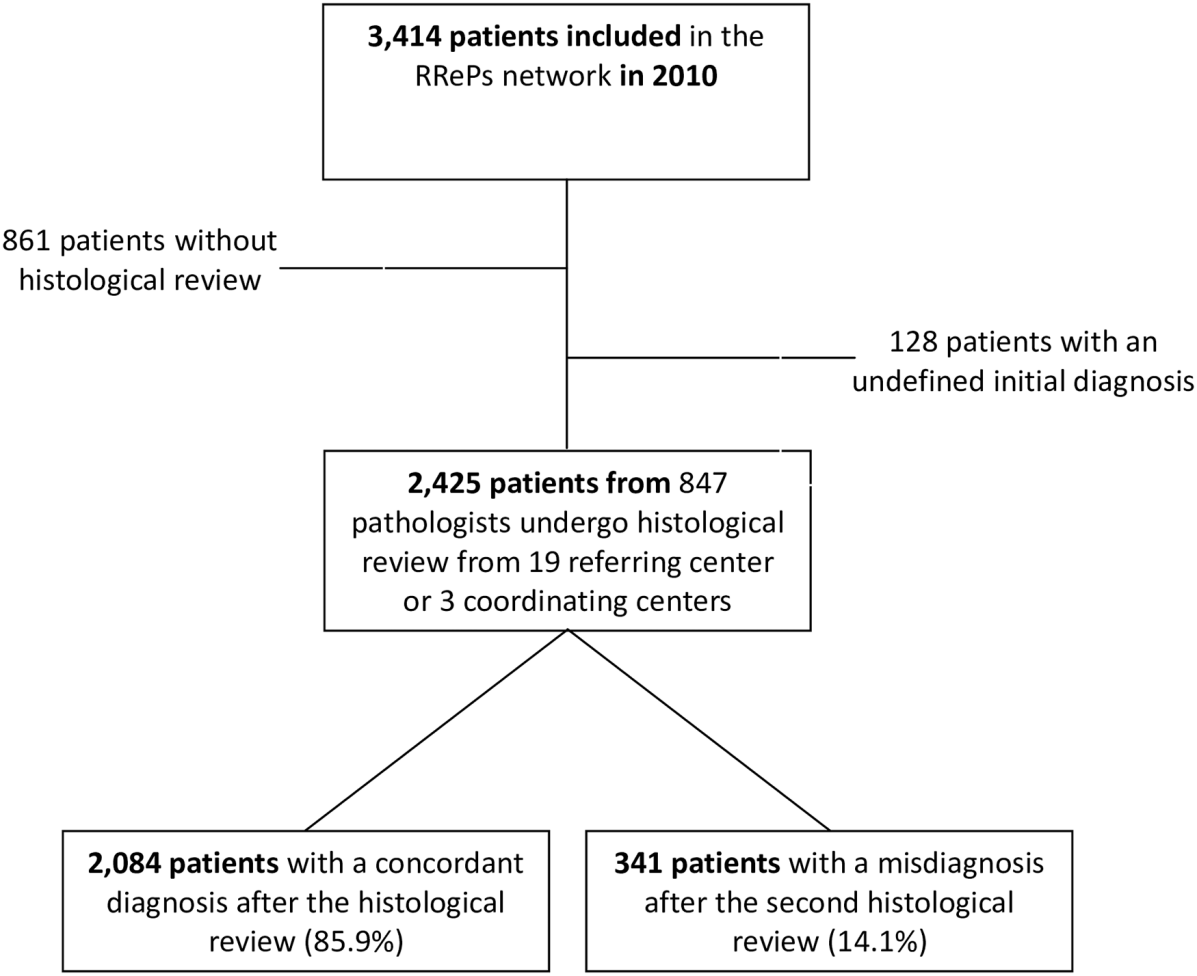
This is the study population.

Of the 3,414 patients included in the RRePS network in 2010, 989 patients were excluded from the study. Of these 989 patients, a histological review was lacking for 861, while 128 patients had an undefined initial diagnosis (Fig 1). Therefore, a total of 2,425 patients were ultimately included in this study to examine the types of discordance that occurred and to identify predictive factors. A total of 2,084 patients (86%) had a concordant diagnosis, whereas 341 patients (14%) had a discordant diagnosis according to the 2013 WHO Classification of Tumors of Soft Tissue and Bone.

### Patient and tumor characteristics

The characteristics of the patients and the tumors are detailed in Tables 1 and 2, respectively. Univariate analyses revealed that the rate of discordant diagnoses was related to the tumor site, tumor size, tumor depth, histological type, condition of review (i.e., a second opinion versus systematic review), and use of molecular biology.

**Table 1:** Patient characteristics.

	Total N = 2425	Concordant diagnoses N = 2,084	Discordant diagnoses N = 341	p-value^a^
***Age (years)***				
Median (range)	62 (1–102)	62 (1–98)	59 (2–102)	0.1490
***Gender***				
Female, n (%)	1,206	1,022 (84.7%)	184 (15.3%)	0.1018
Male, n (%)	1,219	1,062 (87.1%)	157 (12.9%)	
***Tumor site***				**< 0.0001**
Head and neck, n (%)	241	198 (82.2%)	43 (17.8%)	
Trunk wall, n (%)	421	361 (85.8%)	60 (14.2%)	
Internal trunk, n (%)	402	339 (84.3%)	63 (15.7%)	
Lower limb, n (%)	449	401 (89.3%)	48 (10.7%)	
Upper limb, n (%)	220	176 (80.0%)	44 (20.0%)	
Gastrointestinal tract, n (%)	384	350 (91.2%)	34 (8.8%)	
Gynecological area, n (%)	132	120 (90.9%)	12 (9.1%)	
Other viscera, n (%)	174	138 (79.3%)	36 (20.7%)	
Unknown, n (%)	2	1 (50.0%)	1 (50.0%)	
***Tumor size*, *mm***^b^				**< 0.0001**
Median (range)	60 (3–500)	60 (3–500)	40 (5–400)	
***Localization***^c^				**0.0329**
Superficial, n (%)	565	464(82.1%)	101 (17.9%)	
Deep, n (%)	1,571	1,361(86.6%)	210 (13.4%)	
Superficial and deep, n (%)	144	122 (84.7%)	22 (15.3%)	
***Histological type*** ^d^				**< 0.0001**
Sarcoma, n (%)	1,520	1,456 (95.8%)	64 (4.2%)	
*Liposarcoma*	*306*	*290 (94*.*8%)*	*16 (5*.*2%)*	
*Leiomyosarcoma*	*236*	*228 (96*.*6%)*	*8 (3*.*4%)*	
*Myxofibrosarcoma*	*96*	*94 (97*.*9%)*	*2 (2*.*1%)*	
*Rhabdomyosarcoma*	*84*	*84 (100%)*	*0 (0%)*	
*Synovial sarcoma*	*54*	*54 (100%)*	*0 (0%)*	
*MPNST*	*41*	*39 (95*.*1%)*	*2 (4*.*9%)*	
*Kaposi sarcoma*	*49*	*48 (98*.*0%)*	*1 (2*.*0%)*	
*Angiosarcoma*	*75*	*73 (97*.*3%)*	*2 (2*.*7%)*	
*Malignant solitary fibrous tumor*	*29*	*25(86*.*2%)*	*4 (13*.*8%)*	
*Ewing sarcoma*	*21*	*20(95*.*2%)*	*1 (4*.*8%)*	
*Undifferentiated sarcoma*	*393*	*369(93*.*9%)*	*24 (6*.*1%)*	
*Others*^e^	*136*	*132(97*.*1%)*	*4 (2*.*9%)*	
GIST, n (%)	332	326 (98.2%)	6 (1.8%)	
Desmoid tumor, n (%)	123	100 (81.3%)	23 (18.7%)	
Intermediate malignant tumor, n (%)	218	202 (92.7%)	16 (7.3%)	
*Dermatosarcoma protuberans*	*120*	*110 (91*.*7%)*	*10 (8*.*3%)*	
*Others*	*98*	*92 (93*.*9%)*	*6 (6*.*1%)*	
Non-sarcoma malignant tumor, n (%)	85	0 (0%)	85 (100%)	
*Carcinoma*	*53*	*0 (0%)*	*53 (100%)*	
*Melanoma*	*12*	*0 (0%)*	*12 (100%)*	
*Others*	*20*	*0 (0%)*	*20 (100%)*	
Benign tumor, n (%)	147	0 (0%)	147 (100%)	

^**a**^p-values were calculated using Chi-square tests (except for age and tumor size, in which case a Mann-Whitney-Wilcoxon test was used).

^b^Missing data n = 464.

^c^Missing data n = 145.

^***d***^Final diagnosis.

^***e***^Adenosarcoma, chondrosarcoma, sclerosing epithelioid fibrosarcoma, epithelioid hemangioendothelioma, malignant mesenchymoma, osteosarcoma, clear-cell sarcoma, alveolar soft part sarcoma, undifferentiated endometrial sarcoma, epithelioid sarcoma, low-grade fibromyxoid sarcoma, intimal sarcoma, desmoplastic small-round-cell tumor, malignant granular cell tumor, extrarenal rhabdoid tumor, and endometrial stromal sarcoma.

Abbreviations: MPNST: Malignant Peripheral Nerve Sheath Tumors; PNET: Primitive Neuroectodermal Tumor; NOS: Not Otherwise Specified; GIST: Gastrointestinal Stromal Tumor.

**Table 2:** Tumor sample characteristics.

	Total N = 2,425	Concordant diagnoses N = 2,084	Discordant diagnoses N = 341	p-value^a^
***Type of tumor sample***				**0.0430**
Surgical biopsy, n (%)	424	362 (85.4%)	62 (14.6%)	
Core needle biopsy, n (%)	357	322 (90.2%)	35 (9.8%)	
Tumor resection, n (%)	1,644	1,400 (85.2%)	244 (14.8%)	
***Type of secondary review***				**< 0.0001**
Second opinion, n (%)	1,213	952 (78.5%)	261 (21.5%)	
Systematic review, n (%)	845	782 (92.5%)	63 (7.4%)	
Internal Network review, n (%)	367	350 (95.4%)	17 (4.6%)	
**IHC**^b^				**0.0358**
Yes, n (%)	2,313	1,980 (85.6%)	333 (14.4%)	
No, n (%)	110	102 (92.7%)	8 (7.3%)	
**Molecular biology**^c^				**< 0.0001**
Yes, n (%)	665	606 (91.1%)	59 (8.9%)	
No, n (%)	1,742	1,461 (83.9%)	281 (16.1%)	
**FISH**^d^				0.3497
Yes, n (%)	753	654 (86.9%)	99 (13.1%)	
No, n (%)	1,653	1,412 (85.4%)	241 (14.6%)	

^a^p-values were calculated using Chi-square tests.

^b^Missing data n = 2.

^c^ missing data n = 18.

^d^ missing data n = 19.

Abbreviations: IHC: Immunohistochemistry; FISH: Fluorescent in situ hybridization

### Concordance and discordance analysis

Table 3 shows that more than two-thirds (n = 341) of the discordances were in regard to the following: (i) a benign tumor diagnosed as a sarcoma (n = 124/341); (ii) a non-sarcoma malignant tumor diagnosed as a sarcoma (n = 77/341); and (iii) a sarcoma diagnosed as a benign tumor (n = 34/341). We found that 31% of the benign tumors diagnosed as a sarcoma were lipomas (n = 38), while 11% were fasciitis (n = 14). Moreover, 64% of the non-sarcoma malignant tumors diagnosed as a sarcoma were carcinomas (n = 49), and 16% were melanomas (n = 12). Table 4 shows that the patient and the tumor characteristics were not significantly different in terms of the type of histological review (i.e., a second opinion, systematic review, or internal network).

**Table 3:** Number of concordant and misdiagnoses according to the category of tumor (n = 2,425).

Final diagnosis	Sarcoma	Intermediate Malignant Tumor	GIST	Desmoid tumor	Non-sarcoma Malignant tumor	Benign tumor	Total
Initial diagnosis							
Sarcoma	1,456 (87.0%)	6 (0.4%)	5 (0.3%)	5 (0.3%)	77 (4.6%)	124 (7.4%)	**1,673 (69.0%)**
Intermediate Malignant tumor	5 (2.3%)	202 (92.7%)	0 (0%)	1 (0.5%)	3 (1.4%)	7 (3.2%)	**218 (9.0%)**
GIST	14 (3.9%)	0 (0%)	326 (90.6%)	7 (1.9%)	4 (1.1%)	9 (2.5%)	**360 (14.8%)**
Desmoid tumor	1 (0.9%)	0 (0%)	0 (0%)	100 (92.6%)	0 (0%)	7 (6.5%)	**108 (4.5%)**
Non-sarcoma Malignant tumor	10 (100%)	0 (0%)	0 (0%)	0 (0%)	0 (0%)	(0%)	**10 (0.4%)**
Benign tumor	34 (60.7%)	10 (17.9%)	1 (1.8%)	10 (17.9%)	1 (1.8%)	0 (0%)	**56 (2.3%)**
**Total**	**1,520 (62.7%)**	**218 (9.0%)**	**332 (13.7%)**	**123 (5.1%)**	**85 (3.5%)**	**147 (6.1%)**	**2,425 (100%)**

**Table 4:** Patient and tumor characteristics according to the type of histological review (n = 341).

	Second Opinion N = 261	Systematic Review N = 17	Internal Network Review N = 63	p-Value^a^
***Age at diagnosis (years)***				
Median (range)	59 (2.06–101.62)	63 (43–80)	58 (18–92)	0.4081
**Soft tissue**	197 (75.5%)	14 (82.4%)	48 (76.2%)	0.8125
**Viscera**	64 (24.5%)	3 (17.6%)	15 (29.8%)	
***Tumor size*, *mm***^b^				
Median (range)	40 (5–400)	57 (15–160)	40 (7–200)	0.4481
***Localization***^c^				0.1660
Superficial	81 (31.6%)	2 (11.8%)	18 (30.0%)	
Deep	161 (62.9%)	12 (70.6%)	37 (61.7%)	
Superficial and deep	14 (5.5%)	3 (17.6%)	5 (8.3%)	
***Histological type***				0.3975
Sarcoma	53 (20.3%)	3 (17.6%)	8 (12.7%)	
GIST	4 (1.5%)	0 (0%)	2 (3.2%)	
Desmoid tumor	17 (6.5%)	0 (0%)	6 (9.5%)	
Intermediate malignant tumor	12 (4.6%)	1 (5.9%)	3 (4.8%)	
Non-sarcoma malignant tumor	58 (22.2%)	8 (47.1%)	19 (30.2%)	
Benign tumor	117 (44.9%)	5 (29.4%)	25 (39.6%)	
***Type of tumor sample***				0.1752
Surgical biopsy	52 (19.9%)	2 (11.8%)	8 (12.7%)	
Core needle biopsy	27 (10.4%)	4 (23.5%)	4 (6.3%)	
Tumor resection	182 (69.7%)	11 (64.7%)	51 (81.0%)	
**IHC**				0.578
Yes	253 (96.9%)	17 (100%)	63 (100%)	
No	8 (3.1%)	0 (0%)	0 (0%)	
**Molecular biology**^d^				0.963
Yes	46 (17.7%)	3 (17.6%)	10 (15.9%)	
No	214 (82.3%)	14 (82.4%)	53 (84.1%)	
**FISH**^e^				0.173
Yes	76 (29.2%)	8 (47.1%)	15 (23.8%)	
No	184 (70.8%)	9 (52.9%)	48 (76.2%)	

^a^p-values were calculated using Chi-square tests (except for age and tumor size, for which a Mann-Whitney-Wilcoxon test was used).

^b^Missing data n = 65.

^c^Missing data n = 8.

^d^Missing data n = 1.

^e^Missing data n = 1.

Abbreviations: IHC: Immunohistochemistry; FISH: Fluorescent in situ hybridization

### Factors affecting the probability of discordance

As shown in Table 5, multivariate analyses indicated that both the histological type and the type of histological review (i.e., a second opinion *vs*. systematic review) were significant predictors of discordances with sarcomas, GIST, and desmoid tumors. Indeed, the probability of discordance increased when a second opinion was required by the pathologist (OR = 10.5, 95%CI: [3.7; 29.1], p<0.001) or when a systematic review was conducted (OR = 3.1, 95% CI: [1.0; 9.2]; p = 0.046) instead of an internal network review. In addition, discordances were more frequent with desmoid tumors as compared to liposarcomas (OR = 5.1; 95%CI: [2.5; 10.4]; p<0.001) and with liposarcomas as compared to other sarcomas (OR: 0.3; 95%CI: [0.1; 0.7]; p = 0.0168) (Table 5). Finally, the patient characteristics (e.g., age at diagnosis, gender) and the size, location, and type of tumor sample were found to not be significant.

**Table 5:** Predictive factors of discordance with sarcoma, GIST, and desmoid tumors (results of the backward logistic regression analysis).

	Adjusted OR [95%CI]	p-value
**Histological type**^a^ (***Ref = Liposarcoma)***		
Leiomyosarcoma	0.780 [0.325–1.875]	0.5790
Myxofibrosarcoma	0.352 [0.079–1.568]	0.1706
MPNST	0.869 [0.190–3.984]	0.8569
Undifferentiated sarcoma	1.254 [0.649–2.424]	0.5003
GIST	0.385 [0.147–1.005]	0.0512
Desmoid tumor	5.147 [2.554–10.372]	**< 0.0001**
Intermediate malignant tumor	1.559 [0.754–3.222]	0.2308
Kaposi sarcoma	0.353 [0.045–2.740]	0.3190
Angiosarcoma	0.649 [0.144–2.926]	0.5737
Malignant solitary fibrous tumor/ hemangiopericytoma	2.541 [0.774–8.344]	0.1243
Ewing / PNET	0.785 [0.097–6.321]	0.8200
Other sarcoma^b^	0.257 [0.085–0.783]	**0.0168**
**Type of histological review *(Ref = internal network review)***		
Second opinion	10.452 [3.751–29.124]	**< 0.0001**
Systematic review	3.068 [1.018–9.252]	**0.0465**
**No. of observations**	2,193	

^a^Final diagnosis.

^**b**^Adenosarcoma, chondrosarcoma, sclerosing epithelioid fibrosarcoma, epithelioid hemangioendothelioma, malignant mesenchymoma, osteosarcoma, clear-cell sarcoma, alveolar soft part sarcoma, undifferentiated endometrial sarcoma, epithelioid sarcoma, low-grade fibromyxoid sarcoma, intimal sarcoma, desmoplastic small-round-cell tumor, malignant granular cell tumor, extrarenal rhabdoid tumor, endometrial stromal sarcoma.

Abbreviations: MPNST: Malignant Peripheral Nerve Sheath Tumors; PNET: Primitive Neuroectodermal Tumor; NOS: Not Otherwise Specified; GIST: Gastrointestinal Stromal Tumor.

### Cost analysis

**Fig 2:**
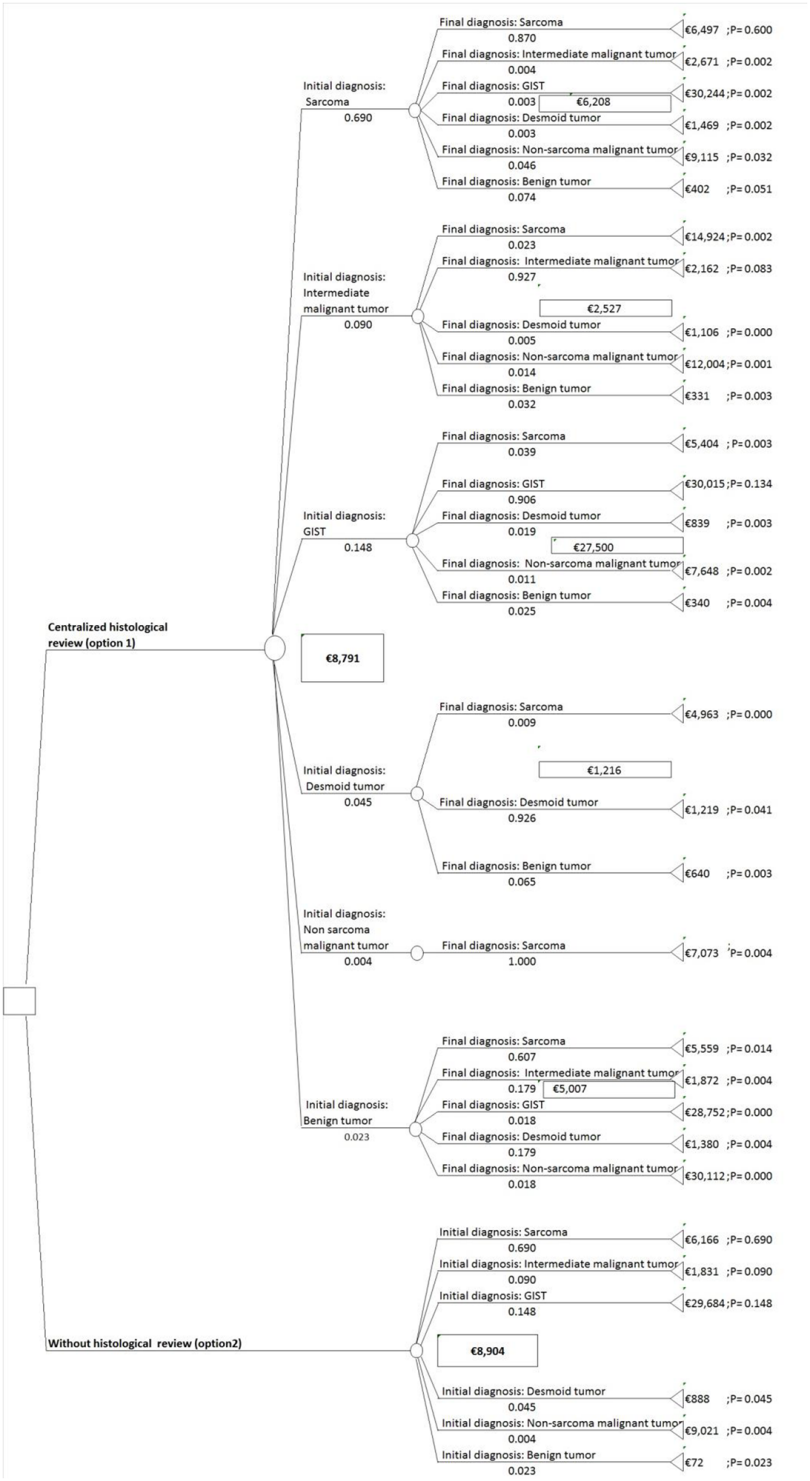
This is the decision tree.

The decision tree for the cost analysis is presented in Fig 2. The square decision node indicates the two options, i.e., disease management based on revised diagnoses after centralized histological review (option 1) or disease management based on diagnoses before review (i.e., without a histological review, referred to as option 2). All mutually exclusive pathways are reported.

- Identification of the resources used and the cost assessment (steps 1 and 2): Of the 341 patients with a discordant diagnosis identified after a histological review, ten patients had insufficient data to establish a disease management process and they were hence excluded from the cost assessment. As an example, the cost assessment for the patient with ID number 16 (initial diagnosis of sarcoma / final diagnosis of GIST) is outlined in S2 File. The main unit costs and the prices used for the pathway calculations are reported in Table 6. The costs are detailed in Table 7.
- Probability calculations (step 3): The estimated probabilities based on the number of concordant and misdiagnoses according to the category of tumor are derived from Table 3. Moving left to right on the decision tree, the first probabilities showed the probabilities of the initial diagnosis for each category of tumor (e.g. the probability of having an initial diagnosis of sarcoma was 0.69). Subsequent probabilities are conditional (e.g. the probability of having a final diagnosis of sarcoma given that initial diagnosis was also sarcoma was 0.87).
- Expected cost calculations in the base-case scenario (step 4): For both options, the expected costs per patient were calculated based on the summation of the pathway costs weighted by the pathway probabilities (obtained by multiplying probabilities along each pathway). As shown by the decision tree, in the base-case scenario, the expected cost per patient amounted to €8,791 when the disease management was based on revised diagnoses after centralized histological review (option 1) and €8,904 when the disease management was based on diagnoses before review (option 2).
- Uncertainty assessment (step 5): Parameters used in the sensitivity analysis for the low and high scenarios are reported in Table 8. As shown in Table 9, for the low and high cost estimate scenarios, the expected cost per patient amounted to €7,033 and €10,549, respectively, when the disease management was based on revised diagnoses after centralized histological review (option 1) versus €7,057 and €10,750, respectively, when the disease management was based on diagnoses before review (option 2).

**Table 6:** Main unitary costs and prices (in € for 2013).

Procedure	Value	Source or code
Thoracic CT scan	€125.78	ZBQK001
Abdominal-pelvic CT scan	€151.35	ZCQK001
Thoracic-abdominal-pelvic CT scan	€176.32	ZZQK024
Whole body CT scan	€176.32	ZZQH033
Cervical-thoracic CT scan	€176.32	ZZQH033
Whole body PET scan	€1,089.54	ZZQL016
Cerebral PET scan	€1,089.54	ACQL002
Pelvic magnetic resonance imaging	€261.34	ZCQN002
Abdominal magnetic resonance imaging	€261.34	ZCQN002
Abdominal-pelvic magnetic resonance imaging	€261.34	ZCQN001
Upper limb magnetic resonance imaging	€261.34	MZQN001
Lower limb magnetic resonance imaging	€261.34	NZQN001
Thoracic magnetic resonance imaging	€261.34	ZBQN001
Soft tissue neck magnetic resonance imaging	€261.34	LCQN001
Head magnetic resonance imaging	€261.34	LAQN001
Cerebral magnetic resonance imaging	€261.34	ACQN001
Pulmonary radiography	€21.28	ZBQK002
Bone scintigraphy (in one session)	€174.58	PAQL00x
Bone scintigraphy (as several session)	€260.13	PAQL00x
Abdominal ultrasound	€56.7	ZCQM008
Eso-gastro-duodenal endoscopy	€96	HEQE002
Functional respiratory investigations	€37.88	GLPQ012
Functional respiratory investigations (with gas measurement)	€67.2	GLPQ013
Myelogram	€9.6	FDHB001
Bronchial fibroscopy	€96	GEQE007
Unilateral Mammography	€37.26	QEQK005
Bilateral Mammography	€66.42	QEQK001
Larynx, and trachea fiberoptic endoscopy	€44.61	GDQE004
Surgery	€1,369.51	DRG 09C101 Other procedures on skin, subcutaneous tissues or breasts, level 1
	€1,543.5	DRG 08C461 Other procedures on soft tissues, level 1
Placement of an implantable venous access device for chemotherapy	€792.52	DRG 05K14Z Management of vascular access (CMD05), length of stay less than 2 days
Chemotherapy (per session)	€395.37	DRG 28z07z Chemotherapy for tumor, per session
Complete hospitalization for chemotherapy	€2,293.91	DRG 17m061 Chemotherapy for other tumor, level 1
External consultation	€18.60	The "Classification Commune des Actes Médicaux" (CCAM)
Imatinib (400 milligrams, 30 tablets)	€2,309.69	French Technical Agency of Information on Hospitals (ATIH)
RRePS network organization (per patient)	€103	Annual budget given by the French National Cancer Institute (2010)
Histological review (per patient)	€228	Lapeyre et al. [6]

**Table 7:** Costs of disease managements (in € for 2013)^a^.

Phases of treatment	Work-up for tumor extension	Surgery	Chemo-therapy	Radio-therapy	Other treatment	Post-treatment surveillance	RRePS network organization and histological review^b^	Total
ID Sarcoma / FD Sarcoma								
Mean (%)	216 (3%)	2,033 (31%)	1,389 (21%)	1,957 (31%)	0 (0%)	570 (9%)	331 (5%)	6,497 (100%)
Min-Max	0–529	0–8,536	0–28,319	0–6,177	0–0	37–1,789	331–331	595–38,711
ID Sarcoma / FD Intermediate malignant tumor								
Mean (%)	68 (3%)	1,830 (69%)	0 (0%)	0 (0%)	0 (0%)	442 (16%)	331 (12%)	2,671 (100%)
Min-Max	0–271	0–3,565	0–0	0–0	0–0	126–560	331–331	890–4,708
ID Sarcoma / FD GIST								
Mean (%)	195 (1%)	1,493 (5%)	27,716 (91%)	0 (0%)	0 (0%)	510 (2%)	331 (1%)	30,244 (100%)
Min-Max	195–195	0–3,903	27,716–27,716	0–0	0–0	510–510	331–331	28,752–32,654
ID Sarcoma / FD Desmoid tumor								
Mean (%)	70 (5%)	386 (26%)	0 (0%)	0 (0%)	107 (7%)	575 (39%)	331 (23%)	1,469 (100%)
Min-Max	0–280	0–1,544	0–0	0–0	0–215	340–840	331–331	885–3,209
ID Sarcoma / FD Non-sarcoma malignant tumor								
Mean (%)	634 (7%)	1,707 (19%)	4,043 (44%)	1,770 (19%)	0 (0%)	630 (7%)	331 (4%)	9,115 (100%)
Min-Max	0–1,407	0–7,998	0–26,428	0–6,694	0–0	37–4,433	331–331	368–32,903
ID Sarcoma / FD Benign tumor								
Mean (%)	7 (2%)	60 (15%)	0 (0%)	0 (0%)	0 (0%)	4 (1%)	331 (82%)	402 (100%)
Min-Max	0–280	0–2,569	0–0	0–0	0–0	0–280	331–331	331–2,900
ID Intermediate malignant tumor / FD Sarcoma								
Mean (%)	374 (3%)	4,245 (28%)	5,664 (38%)	3,430 (23%)	0 (0%)	880 (6%)	331 (2%)	14,924 (100%)
Min-Max	144–529	3,565–6,965	0–28,319	0–6,177	0–0	585–1,169	331–331	4,903–38,710
ID Intermediate malignant tumor / FD Intermediate malignant tumor								
Mean (%)	81 (4%)	1,514 (70%)	0 (0%)	0 (0%)	0 (0%)	236 (11%)	331 (15%)	2,162 (100%)
Min-Max	0–280	0–3,565	0–0	0–0	0–0	37–560	331–331	411–4,709
ID Intermediate malignant tumor / FD Desmoid tumor								
Mean (%)	0 (0%)	0 (0%)	0 (0%)	0 (0%)	215 (19%)	560 (51%)	331 (30%)	1,106 (100%)
Min-Max	0–0	0–0	0–0	0–0	215–215	560–560	331–331	1,106–1,106
ID Intermediate malignant tumor / FD Non-sarcoma malignant tumor								
Mean (%)	97 (1%)	3,619 (30%)	7,278 (60%)	0 (0%)	0 (0%)	679 (6%)	331 (3%)	12,004 (100%)
Min-Max	0–195	0–7,238	0–14,556	0–0	0–0	37–1,322	331–331	368–23,641
ID Intermediate malignant tumor / FD Benign tumor								
Mean (%)	0 (0%)	0 (0%)	0 (0%)	0 (0%)	0 (0%)	0 (0%)	331 (100%)	331 (100%)
Min-Max	0–0	0–0	0–0	0–0	0–0	0–0	331–331	331–331
ID GIST / FD Sarcoma								
Mean (%)	191 (4%)	1,360 (25%)	2,447 (45%)	412 (8%)	0 (0%)	663 (12%)	331 (6%)	5,404 (100%)
Min-Max	144–195	0–8,536	0–14,556	0–6,177	0–0	390–780	331–331	1,110–15,861
ID GIST / FD GIST								
Mean (%)	191 (1%)	1,245 (4%)	27,710 (92%)	0 (0%)	0 (0%)	538 (2%)	331 (1%)	30,015 (100%)
Min-Max	170–195	0–3,903	27,680–27,716	0–0	0–0	510–680	331–331	28,752–32,655
ID GIST / FD Desmoid tumor								
Mean (%)	28 (3%)	0 (0%)	0 (0%)	0 (0%)	92 (11%)	388 (46%)	331 (40%)	839 (100%)
Min-Max	0–195	0–0	0–0	0–0	0–215	340–510	331–331	670–1,250
ID GIST / FD Non-sarcoma malignant tumor								
Mean (%)	595 (8%)	2,462 (32%)	3,386 (44%)	0 (0%)	0 (0%)	874 (12%)	331 (4%)	7,648 (100%)
Min-Max	195–1,284	0–12,312	0–14,556	0–0	0–0	780–972	331–331	1,497–16,861
ID GIST / FD Benign tumor								
Mean (%)	0 (0%)	0 (0%)	0 (0%)	0 (0%)	0 (0%)	9 (3%)	331 (97%)	340 (100%)
Min-Max	0–0	0–0	0–0	0–0	0–0	0–75	331–331	331–406
ID Desmoid tumor / FD Sarcoma								
Mean (%)	144 (3%)	3,903 (78%)	0 (0%)	0 (0%)	0 (0%)	585 (12%)	331 (7%)	4,963 (100%)
Min-Max	144–144	3,903–3,903	0–0	0–0	0–0	585–585	331–331	4,963–4,963
ID Desmoid tumor / FD Desmoid tumor								
Mean (%)	57 (5%)	127 (11%)	0 (0%)	0 (0%)	140 (11%)	564 (46%)	331 (27%)	1,219 (100%)
Min-Max	0–280	0–1,544	0–0	0–0	0–215	340–840	331–331	671–3,209
ID Desmoid tumor / FD Benign tumor								
Mean (%)	0 (0%)	309 (48%)	0 (0%)	0 (0%)	0 (0%)	0 (0%)	331 (52%)	640 (100%)
Min-Max	0–0	0–1,544	0–0	0–0	0–0	0–0	331–331	331–1,874
ID Non-sarcoma malignant tumor / FD Sarcoma								
Mean (%)	185 (3%)	2,030 (29%)	2,009 (28%)	1,681 (23%)	0 (0%)	838 (12%)	331 (5%)	7,073 (100%)
Min-Max	144–195	0–5,852	0–14,556	0–6,177	0–0	160–1,789	331–331	915–27,028
ID Benign tumor / FD Sarcoma								
Mean (%)	215 (4%)	1,946 (35%)	74 (1%)	2,599 (47%)	0 (0%)	394 (7%)	331 (6%)	5,559 (100%)
Min-Max	0–456	0–5,519	0–2,374	6,177	0–0	37–877	331–331	595–12,840
ID Benign tumor / FD Intermediate malignant tumor								
Mean (%)	92 (5%)	1,309 (70%)	0 (0%)	0 (0%)	0 (0%)	140 (7%)	331 (18%)	1,872 (100%)
Min-Max	0–280	0–1,837	0–0	0–0	0–0	37–560	331–331	410–2,434
ID Benign tumor / FD GIST								
Mean (%)	195 (1%)	0 (0%)	27,716 (96%)	0 (0%)	0 (0%)	510 (2%)	331 (1%)	28,752 (100%)
Min-Max	195–195	0–0	27,716–27,716	0–0	0–0	510–510	331–331	28,752–28,752
ID Benign tumor / FD Desmoid tumor								
Mean (%)	76 (6%)	125 (9%)	0 (0%)	0 (0%)	176 (13%)	672 (48%)	331 (24%)	1,380 (100%)
Min-Max	0–280	0–1,370	0–0	0–0	0–215	433–840	331–331	872–2,457
ID Benign tumor / FD Non-sarcoma malignant tumor								
Mean (%)	1,108 (4%)	0 (0%)	26,428 (88%)	1,523 (5%)	0 (0%)	722 (2%)	331 (1%)	30,112 (100%)
Min-Max	1,108–1,108	0–0	26,428–26,428	1,523–1,523	0–0	722–722	331–331	30,112–30,112
ID Sarcoma								
Mean (%)	216 (4%)	2,034 (33%)	1,389 (23%)	1,957 (31%)	0 (0%)	570 (9%)	N/A	6,166 (100%)
Min-Max	0–529	0–8,536	0–28,319	0–6,177	0–0	37–1,789	N/A	264–38,380
ID Intermediate malignant tumor								
Mean (%)	81 (4%)	1,514 (83%)	0 (0%)	0 (0%)	0 (0%)	236 (13%)	N/A	1,831 (100%)
Min-Max	0–280	0–3,565	0–0	0–0	0–0	37–560	N/A	80–4,378
ID GIST								
Mean (%)	191 (1%)	1,245 (4%)	27,710 (93%)	0 (0%)	0 (0%)	538 (2%)	N/A	29,684 (100%)
Min-Max	170–195	0–3,903	27,680–27,716	0–0	0–0	510–680	N/A	28,421–32,324
ID Desmoid tumor								
Mean (%)	57 (6%)	127 (14%)	0 (0%)	0 (0%)	140 (16%)	564 (64%)	N/A	888 (100%)
Min-Max	0–280	0–1,544	0–0	0–0	0–215	340–840	N/A	341–2,878
ID Non-sarcoma malignant tumor								
Mean (%)	624 (7%)	1,779 (20%)	4,335 (48%)	1,616 (18%)	0 (0%)	647 (7%)	N/A	9,021 (100%)
Min-Max	0–1,407	0–12,312	0–26,428	0–6,694	0–0	37–4,433	N/A	37–32,572
ID Benign tumor								
Mean (%)	6 (8%)	62 (86%)	0	4 (6%)	0	0	N/A	72
Min-Max	0–280	0–2,569	0–0	0–280	0	0	N/A	0–2,569

^a^Based on hypothetical resources used according to the characteristics and disease of the patients from the RRePS network (2010) and using national and international guidelines. Due to budgetary constraints, the costs of disease management for patients with a concordant diagnosis were estimated from the final diagnosis of the patients with a discordant diagnosis, whose sample size was smaller than the concordant cases.

^b^Cost of RRePS network organization (€102 per patient) and Histological review (€228 per patient) included.

Abbreviations: ID: initial diagnosis, FD: final diagnosis, and N/A: not applicable.

**Table 8:** Costs parameters of disease management used in the decision-tree model (in € for 2013).

Cost parameter	Base-case^a^	High cost estimate (base-case +20%)	Low cost estimate (base-case -20%)
ID Sarcoma / FD Sarcoma	€6,497	€7,796	€5,197
ID Sarcoma / FD Intermediate malignant tumor	€2,671	€3,205	€2,136
ID Sarcoma / FD GIST	€30,244	€36,292	€24,195
ID Sarcoma / FD Desmoid tumor	€1,469	€1,762	€1,175
ID Sarcoma / FD Non-sarcoma malignant tumor	€9,115	€10,937	€7,292
ID Sarcoma / FD Benign tumor	€402	€483	€322
ID Intermediate malignant tumor / FD Sarcoma	€14,924	€17,909	€11,940
ID Intermediate malignant tumor / FD Intermediate malignant tumor	€2,162	€2,594	€1,729
ID Intermediate malignant tumor / FD Desmoid tumor	€1,106	€1,327	€884
ID Intermediate malignant tumor / FD Non-sarcoma malignant tumor	€12,004	€14,405	€9,604
ID Intermediate malignant tumor / FD Benign tumor	€331	€351	€310
ID GIST / FD Sarcoma	€5,404	€6,484	€4,323
ID GIST / FD GIST	€30,015	€36,017	€24,012
ID GIST / FD Desmoid tumor	€839	€1,006	€671
ID GIST / FD Non-sarcoma malignant tumor	€7,648	€9,178	€6,119
ID GIST / FD Benign tumor	€340	€407	€272
ID Desmoid tumor /FD Sarcoma	€4,963	€5,955	€3,970
ID Desmoid tumor / FD Desmoid tumor	€1,219	€1,462	€975
ID Desmoid tumor / FD Benign tumor	€640	€767	€512
ID Non-sarcoma malignant tumor / FD Sarcoma	€7,073	€8,488	€5,659
ID Benign tumor / FD Sarcoma	€5,559	€6,671	€4,448
ID Benign tumor / FD Intermediate malignant tumor	€1,872	€2,246	€1,497
ID Benign tumor / FD GIST	€28,752	€34,502	€23,001
ID Benign tumor / FD Desmoid tumor	€1,380	€1,655	€1,104
ID Benign tumor / FD Non-sarcoma malignant tumor	€30,112	€36,134	€24,089
ID Sarcoma	€6,166	€7,465	€4,868
ID Intermediate malignant tumor	€1,831	€2,263	€1,398
ID GIST	€29,684	€35,687	€23,681
ID Desmoid tumor	€888	€1,132	€644
ID Non-sarcoma malignant tumor	€9,021	€10,891	€7,151
ID Benign tumor	€72	€86.4	€57.6

^**a**^Based on hypothetical resources used according to the characteristics and disease of the patients from the RRePS network (2010) and using national and international guidelines. Due to budgetary constraints, the costs of disease management for patients with a concordant diagnosis were estimated from the final diagnosis of the patients with a discordant diagnosis, whose sample size was smaller than the concordant cases.

Abbreviations: ID: initial diagnosis, FD: final diagnosis.

**Table 9:** Expected costs per patient for base-case, high cost, and low cost estimate scenarios (in € for 2013).

Strategy	Base-case	High cost estimate scenario	Low cost estimate scenario
Disease management based on revised diagnoses after centralized histological review (option 1)	€8,791	€10,549	€7,033
Disease management based on diagnoses before review (i.e. without histological review) (option 2)	€8,904	€10,750	€7,057
Cost-saving per patient using option 1 instead of option 2	€113	€201	€24

Additional one-way sensitivity analyses showed that an increase of 20% in the cost of the histological review would increase the expected cost per patient by €21, while an increase of 20% in the cost of the RRePS network organization would increase the cost per patient by €46.

## Discussion

### Histological review procedure within the RRePS network reduces misdiagnoses

This retrospective study using prospectively implemented databases was based on data collected at the national level that reached a coverage rate of 83% [22]. Also, using the criteria established by the 2013 WHO Classification of Tumors of Soft Tissue and Bone, our findings indicate a concordance rate of 86% with sarcomas, GIST, and desmoid tumors. Moreover, the observed discordance rate of 14% is lower than what has been reported in other sarcoma studies [7,11,15,23], thus indicating an overall improvement in the performance of the health care system in this regard in the last 10 years. In fact, discordance rates of 33%, 43%, 46%, and even 66% have been observed when broader definitions of discordance were applied [7, 11, 15, 23]. For example, some studies have taken the grade into consideration when assessing misdiagnoses [11, 15, 23]. However, the histopathological grade and/or subtype differences after histological review were not taken into account in the present study. Notably, our 14% discordance rate is in line with a previous study by Thway and Ray-Coquard that only considered major discrepancies able to change medical management [12, 15]. Cases sent for histological review had a discordance rate of 21.5% for second opinions, while those sent for systematic review had a discordance rate of 7.5%. Indeed, these review processes represent two very different situations. In the first case, the pathologist has a doubt and then requests an expert to review the case, whereas in the second circumstance, the pathologist requires the review to comply with French NCI recommendations. These data are in agreement with reports in the literature regarding second opinions [7, 15, 23] and systematic reviews [12, 15, 23]. On the other hand, Lehnhardt et al. reported a rate of incorrect diagnoses upon second opinion of 2.5% [24]. This discrepancy could be explained by the fact that the histological reviews were performed within a single center in the study by Lehnhardt et al., while our investigation was carried out within the national framework of the 22 pathology centers included within the RRePS network. Also, for the present study, we can assume that the pathology centers of the RRePS network sent the most difficult cases to the pathology coordinating centers.

A discordance rate of 4.6% within the RREPS network review was observed (Table 2). These represent cases selected by the reference centers after having eliminated all of the cases with a specific molecular or immunohistochemical anomaly. Moreover, these cases corresponded to a transitional training process for the referring centers that is no longer followed. The referring or regional centers belonging to RRePS currently review their difficult cases during monthly national meetings or they send these cases directly to one of the coordinating centers.

This study shows that benign tumors diagnosed as a sarcoma represented 37% of the discordances, while non-sarcoma malignant tumors diagnosed as a sarcoma represented 23% of the discordances. These results are in line with a previous study by Arbiser et al., reporting discordance rates of 45% and 20%, respectively [7]. Benign tumors diagnosed as a sarcoma were most likely to be lipomas and fasciitis, and non-sarcoma malignant tumors diagnosed as a sarcoma were often metastatic carcinomas and melanomas. These results are also in agreement with the prior study by Arbiser et al. [7]. Notably, the lesions involved in misdiagnoses are relatively few when compared to the total number of cases sent for histological review. Therefore, it is important to highlight the tumor characteristics that are associated with discordances for pathologists. In this regard, adipocytic tumors or small superficial tumors require particular attention when diagnosing sarcoma, keeping in mind that the gold standard for adipocytic tumors is *MDM2* amplification [25]. Of note, the high discordance rate for desmoid tumors was surprising, although this could be explained by the rarity of this tumor (i.e., 300 new cases per year in France for about 1,400 pathologists).

Our logistic regression analyses yielded the following useful information for pathologists, clinicians, and health policy makers: (i) desmoid tumors have an increased probability of discordance compared to liposarcomas and (ii) sending initial diagnoses for second opinions when diagnostic difficulties are encountered greatly increases the probability of discordance compared to an internal network review. In this regard, discordances should not be viewed as “errors” but as an acknowledged need for assistance. In addition, our analysis revealed that initial diagnoses sent for a systematic review according to the French National Cancer Institute (INCa) recommendations also increased the probability of discordance as compared to a primary analysis within the network.

### Histological review procedure within the RRePS network provides cost savings for the French National Health Insurance

Our results confirm the importance of centralized histological review within specialized networks for sarcomas, GIST, and desmoid tumors. The cost analysis provides useful information to support clinical decision-making to promote appropriate treatment of patients. Our findings suggest that centralized histological review is likely to result in a cost-saving of €113 per patient (including the cost of RRePS network organization and the centralized histological review) in the base-case analysis (€201 and €24 in the high- and low-case cost estimate scenarios, respectively). Even with an increase of 20% in the cost of histological review (i.e., from €228 to €274), centralized histological review still provides a cost-saving of €67 per patient (i.e., €8,904—(€8,791 + €46)). The cost of RRePS network organization and the centralized histological review remain modest compared with the costs incurred by a course of chemotherapy, radiation, or additional surgery.

To the best of our knowledge, this is the first analysis comparing the costs of disease management based on revised diagnoses after histological review and disease management based on diagnoses before review for sarcoma, GIST, and desmoid tumor patients.

### Limitations

This study has several limitations, however:

- The cost assessment was based on theoretical disease management by experts, since the “true” resource consumption for both options remains unknown. Indeed, an extraction from the SNIIRAM (Système National Inter-régime de l’Assurance Maladie), which contains individual, anonymous, and exhaustive data for all health spending reimbursements, will provide a mix of the two options depending on the delay between the initial diagnosis and the histological review [26].
- As the workload related to the systematic reviewing of accounts of anatomopathology and the construction and validation of theoretical disease management was very time consuming, the time horizon was restricted to 12 months. Hence, the economic evaluation was limited to the calculation of incremental costs between options 1 and 2, without taking into account all of the economic consequences of a diagnostic discrepancy. This assumption probably lowers the cost saving per patient for the centralized histological review option, as (i) mid- and long-term direct health care costs (e.g. the cost of relapse, the cost of permanent disability), (ii) direct non-health care costs (e.g. transportation, household activities), and (iii) indirect costs (e.g., productivity loss) were not included in this study. Indeed, studies assessing the costs associated with histological review identified the benefit of initial diagnoses submitted for a histological review before/during the treatment [8, 10, 27, 28]. Thus, a cost-benefit study performed in the 1980s in the United States observed a cost-benefit ratio of 2.63 (i.e., for each dollar spent on the histological review program, there was a savings of $2.63) [10, 29]. Also, an investigation carried out in 2006 found that histological review programs provided cost savings with cases of sarcoma [8].
- Health-related quality of life was not taken into account in this study and, to our knowledge, no cost utility analysis has been performed as yet, as recommended by the National Institute for Health and Clinical Excellence (NICE) [30, 31]. As a diagnostic discrepancy may result in the patient’s death or permanent disability and significant emotional damage, further analyses will need to be performed using a discrete-event simulation model [32] for example. This method of modeling appears to be suitable with sarcoma, GIST, and desmoid tumors for at least three reasons: (i) the characteristics of the patients and their illness(es) (e.g., age, tumor site, histological type, etc.) are heterogeneous; (ii) the formation of a queue, which may affect the patients’ ability to access this histological review procedure within the RRePS network cannot be excluded; (iii) the order in which events occur (i.e., events that require clinicians to make decisions) is important, and potentially has a major impact on patient health conditions and costs.
- The tumor grade was not systematically incorporated into the shared database. As the difficulty in making the diagnosis can depend on the grade, this has an impact on discordant diagnoses with sarcoma [17, 25] and other diseases [7]. However, the relevance of the grade has declined since the development of targeted therapies based on histological subtypes and molecular anomalies.

## Conclusions

Our results should assist with defining health policies regarding the organization of histological review of sarcomas, GIST, and desmoid tumors from both clinical and economic points of view. From a clinical perspective, initial diagnoses sent either for second opinion or systematic review according to the French National Cancer Institute (INCa) recommendations increased the probability of discordance as compared to a primary analysis within the RRePS network. From an economic perspective, centralized histological reviews lower the cost of patient disease management for the French NHI. Moreover, our findings shed light on the overdiagnosis of sarcoma, which was found to most often be confused with benign tumors. Care should also be taken when diagnosing desmoid tumors, as they similarly exhibit high rates of discordance.

## Supporting information

S1 File This is the clinical situation, characteristics of the patient, and hypothetical therapeutic decision questionnaire. (PDF)

S2 File This is the clinical situation, hypothetical therapeutic decision, and cost assessment for the patient with ID number 16 (initial diagnosis sarcoma / final diagnosis GIST). (PDF)
