# Supplementary material for: The cost-saving effect of centralized histological reviews with soft tissue and visceral sarcomas, GIST, and desmoid tumors: The experiences of the pathologists of the French Sarcoma Group
Source: PLoS One. 2018 Apr 5;13(4):e0193330. doi: 10.1371/journal.pone.0193330 (PMC5886412; doi:10.1371/journal.pone.0193330)
Supplement: S1 File — (PDF) [file pone.0193330.s001.pdf]

Clinical situation and characteristics of the patient

1.1. ID number	|__|__|__|__|__|
1.2. Gender	Male ☐ Female ☐
1.3. Age at diagnosis	|__|__|
1.4. Performance Status (OMS)	1☐ 2☐ 3☐ 4☐ 5☐
1.5. Previous cancer	Yes ☐ No ☐ Unknown ☐
1.6. Sarcoma in irradiated tissue	Yes ☐ No ☐ Unknown ☐
1.7. Exact location of the lesion:	....
1.7.1. IF visceral, specify:	Colon ☐ Duodenum ☐ Stomach ☐ Small intestine ☐ Esophagus ☐ Rectum ☐
1.8. Depth of the lesion	Mixed ☐ Deep ☐ Superficial ☐ Unknown ☐
1.9. Clinical size of the tumor (mm):	|__|__|__|
1.10. Stage of the tumor:	Local ☐ Loco-regional ☐ Local and metastatic ☐ Unknown ☐
1.11. Type of tissue sampling	Microbiopsy ☐ Surgical biopsy ☐ Resection ☐
1.12. IF resection, please specify:	R0 ☐ R1 ☐ R2 ☐ Unknown ☐
1.13. Histological diagnosis	______________________________
1.14. IF sarcoma, please specify the grade	1 ☐ 2 ☐ 3 ☐ Not specified ☐
IF GIST, please specify the Miettinen's risk classification:	Low risk ☐ Intermediary risk ☐ High risk ☐ Not specified ☐
IF GIST, please specify the type of mutation:	______________________________
1.15 Molecular biology	Yes ☐ No ☐
IF Yes :	Positive ☐ Non-contributive ☐ Negative ☐
1.16. FISH :	Yes ☐ No ☐
If yes :	Positive ☐ Non-contributive ☐...

Hypothetical therapeutic decision

Work-up for extension	Yes ☐ No ☐
IF yes, please specify the imaging:	Thoracic-Abdominal-Pelvic CT scan ☐ Pulmonary radiology ☐<br>Other (please list):
Surgery:	Yes ☐ No ☐
IF YES	Primary surgery ☐ Re-excision ☐
IF YES, place in the treatment schedule	1 ☐ 2 ☐ 3 ☐
Chemotherapy:	Yes ☐ No ☐
IF Yes, place in the treatment schedule	Adjuvant ☐<br>Neoadjuvant ☐<br>CT neoadjuvant, then surgery, then adjuvant ☐<br>Palliative CT ☐
Chemotherapy protocol:	____________________
Radiotherapy:	Yes ☐ No ☐
IF YES, place in the treatment schedule:	1 ☐ 2 ☐ 3 ☐ Concomitant with CT ☐
If YES, total dose:	50 Gy ☐ 60 Gy ☐ other (please list):____________
Other treatments:	Yes ☐ No ☐
IF YES, please select:	
Post-treatment surveillance (during the first year)	Yes ☐ No ☐
If YES, please select the type of medical examination?	Physical examination ☐ MRI ☐ Thoracic-Abdominal-Pelvic CT scan ☐ Pulmonary radiology ☐<br>Abdominal ultrasound ☐ Other
At what frequency?	Every 3 months ☐ Every 4 months ☐ Every 6 months ☐<br>Every 12 month ☐
Other:	
Given the available information, how would you rate your theoretical therapeutic decision:	Impossible to define ☐ unreliable ☐ Moderately reliable ☐ Very reliable ☐
IF your answer is “unreliable”, please specify the reason(s)?	
