# Supplementary material for: The cost-saving effect of centralized histological reviews with soft tissue and visceral sarcomas, GIST, and desmoid tumors: The experiences of the pathologists of the French Sarcoma Group
Source: PLoS One. 2018 Apr 5;13(4):e0193330. doi: 10.1371/journal.pone.0193330 (PMC5886412; doi:10.1371/journal.pone.0193330)
Supplement: S2 File — (PDF) [file pone.0193330.s002.pdf]

A 55-year-old woman at diagnosis, presented with a histological diagnosis of GIST (Gastrointestinal stromal tumors) after centralized histological review, whereas the initial diagnosis was a leiomyosarcoma, with a high risk according to the Miettinen's risk classification. The molecular biology was positive. The lesion was deep, with a clinical size of 100 mm. The stage of the tumor was both local and metastatic. The type of tissue sampling was a resection (R1). The patient had a previous cancer.

In keeping with the recommendations for clinical practice, and based on the histological diagnosis after centralized histological review, the hypothetical therapeutic decision was to perform:

- A work-up for tumor extension including an external consultation and a thoracic-abdominal-pelvic CT scan. Costs were: €18.6 + €176.32 = €194.92 (see Table 6);
- A chemotherapy treatment based on imatinib 400 milligrams. Costs were €2,309.69×12 months = €27,716.28;
- A post-treatment surveillance including an abdominal-pelvic CT scan associated with an external consultation every four months. Costs were: ((€18.60 + €151.35) × 3 = €509.85);
- Additional costs for RRePS network organization and histological review were (€102.52 + €228 = €330.52)

The cost of the patient with ID number 16 was hence estimated to be €28,751.57 (€194.20 + €27,716.28 + €509.85 + €330.52).
